# Retraction of: KCNQ1OT1 promotes melanoma growth and metastasis

**DOI:** 10.18632/aging.206214

**Published:** 2025-02-28

**Authors:** Bingyu Guo, Qian Zhang, Hongyi Wang, Peng Chang, Kai Tao

**Affiliations:** 1 Department of Reconstructive and Plastic Surgery, The General Hospital of Shenyang Military Region, Shenyang, China

**Keywords:** lncRNA, miRNA, KCNQ1OT1, miR-153, melanoma

**This article has been retracted:** Aging has completed its investigation of this paper. We found multiple instances of image overlap, both internal and external. Specifically:

Figure 6: The KCNQ1OT1 migration image in panel E overlaps with the invasion control image in panel H.

Figure 7: The KCNQ1OT1 image in panel B overlaps with the miR-153 AS image in panel F.

Figure 6C control image overlaps with the si-KCNQ1OT1 + miR-153 AS image in Figure 7F.

Figure 6D reuses transwell assay images from Figures 4A and 5C of the authors earlier published paper [1].

Figures 6C and 7F contain transwell assay images that overlap with images from an unrelated 2017 paper from a different institution [2].

The corresponding author informed us that these overlaps were due to improper data storage in the laboratory and has applied for a retraction. Given these findings, the Editorial decision was to retract the paper. All authors have agreed to this retraction.
